# Calixtyrosol: a Novel Calixarene Based Potent Radical Scavenger

**Published:** 2015

**Authors:** Fazel Nasuhi Pur, Karim Akbari Dilmaghani

**Affiliations:** 1 *Health Technology Incubator Center, Urmia University of Medical Science, Urmia, Iran. *; 2 *Department of Chemistry, Faculty of Science, Urmia University, Urmia, Iran.*

**Keywords:** Calixtyrosol, Calixarene, Tyrosol, Antioxidant, Radical scavenging

## Abstract

The oxidative stress causes many diseases in human, therefore antioxidants have a special position in the medicinal chemistry. Tyrosol is an important antioxidant with a plenty of biological properties. There are many strategies such as clustering single drug units in order to develop new drugs. The cluster effect can increase drug effects relative to single drug unit. Calixtyrosol is the novel cluster of tyrosol that shows a more effective antioxidant activity than single tyrosol. In fact, tyrosol can be considered as 1/4 of the cluster.

Four hydroxyethyl moieties have been grafted at the upper rim of the calix[4]arene in all-*syn* orientation, giving novel agent in the field of antioxidant agents. Free radical scavenging tests were determined by the 2, 2-diphenyl-1-picrylhydrazyl radical in methanol for four antioxidants: calixtyrosol, tyrosol, hydroxytyrosol and 3, 5-di-*tert*-buty l-4-hydroxytoluene to compare their antioxidant activity.

Free radical scavenging test showed that calixtyrosol has enhanced antioxidant activity in comparison to the corresponding single tyrosol unit (> 5 fold), it has even more active than the other test antioxidants (2 fold). Presumably, it is attributed to tethering and arraying of four impacted tyrosol units, which make a synergistic effect in interactions with radicals for creating effective radical scavenging activity.

This method is in debt of synergistic effect, tethering and arraying of single units in the cluster structure.

## Introduction

In recent years, due to a variety of human diseases associated with oxidative stress, there has been special interest in antioxidants in the medicinal chemistry (1-3). In any biological system, a serious balance must be kept up between the formation of radicals ROS (Reactive oxygen species) and RNS (Reactive nitrogen species) and their annihilators. These reactive radicals are products of natural pathways of organs in the human body, but in excessing of their volumes under definite conditions, they can act as dangerous compounds. The free-radical formation process causes to damage and death of cells, accelerates aging and it is the major factor for many diseases such as cancer, cardiovascular and heart diseases (2-6).

The Mediterranean diet, with composition of fatty acids and phenolic antioxidants (i.e., in olive oil), provides greater resistance to oxidative stress, which is the main cause of the diseases is mentioned above (7, 8).

**Figure 1:**
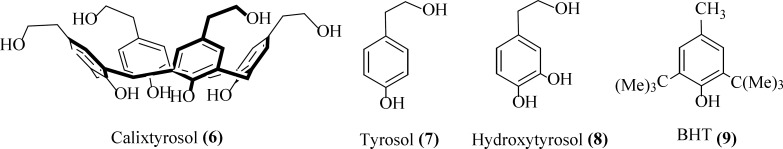
Chemical structures of phenolic antioxidants.

The main olive oil simple phenols are tyrosol and hydroxytyrosol (Figure 1) that have shown antioxidant properties (9), such as: inhibiting peroxidation processes on human low density lipoproteins (LDL) particles in numerous *in-vitro* experiments, contributing to the reduction of ROS-RNS and scavenging NOO¯ and O_2_¯ (2, 10-17).

Tyrosol [2-(4-hydroxyphenyl) ethyl-alcohol] is a liposoluble, noncarboxyl mono-phenol compound, formed during yeast fermentation from tyrosine [3-(4-hydroxyphenyl)-alanine]. In addition to its antioxidative effects (18-20), tyrosol has the neuroprotective (5, 21), anti-inflammatory (22), antiaging (4), and antifungal (23) properties. Moreover, researches have shown that tyrosol possesses the ability to modulate human LDL levels besides having cardioprotector action (24, 25). Also, it is known that tyrosol inhibits lipopolysaccharide (LPS)-induced cytokine releasing from human monocytes (26) and LPS-induced leukotriene B4 releasing in human mononuclear cells (27).

These reasons prompted us to synthesize a compound by using a rigid molecular platform for the demonstration of tyrosol cluster. This idea, could made a molecular building with enhanced effects and radical scavenging activity in comparison to a single tyrosol unit.

Calixarenes have many structural characteristics that are preferable for the design and development of new drugs. Recently, due to calix[4]arene limited toxicity, they have been used in biological field as building blocks or molecular scaffolds (28-32). For medical applications, the toxicity of molecules is evidently a key factor; to date the calixarenes have showed neither toxicity nor immune responses (33, 34).

Herein we wish to report free radical scavenging and antioxidative activity of calix[4]arene derivative possessing four units of tyrosol in all-*syn* orientation.

## Experimental

*General Procedure*

The melting points of all compounds were recorded on Philip Harris C4954718 apparatus without calibration. IR spectra were determined on a Thermo Nicolet 610 Nexus FT-IR spectrometer in KBr disks. UV/visible spectra were recorded on a Hewlett-Packard 8453 diode array spectrometer equipped with a magnetically stirred cell (optical pathlength 1 cm) ^1^H (400 MHz) and ^13^C (100 MHz) NMR measurements were recorded on a Bruker AM-400 spectrometer in DMSO-d_6_ using TMS as the internal reference. Elemental analysis were performed using a Heraeus CHN-O-Rapido analyzer. Mass spectra were recorded on a JEOL-JMS 600 (FAB MS) instrument. Thin layer chromatography (TLC) analyses were carried out on silica gel plates. All chemicals were purchased from Merck, Sigma-Aldrich and Fluka Chemie (Tehran, Iran) and used as received by standard procedures, such as antioxidants and DPPH. All reactions were carried out under a nitrogen or argon atmosphere.

*Chemistry*

*p*-*tert*-Butyl calix[4]arene 1 was prepared according to Gutsche’s method as white crystals (35). Calix[4]arene 2 was prepared by the previously reported method as white powder (36).

*Mannich dimethylaminomethylation for the synthesis of compound 3*

Acetic acid (4.5 mL), 40% aqueous dimethylamine (2.25 g, 20 mmol) and 37% aqueous formaldehyde (1.62 g, 20 mmol) were added to the solution of compound 2 (1.6 g, 4 mmol) in THF (35 mL). The reaction mixture was stirred for 24 h at room temperature, the solvents were removed under vacuum, and the residue was dissolved in of water (25 mL). The aqueous solution was extracted two times with ether (20 mL) and neutralized with 10% K_2_CO_3_ solution, and the precipitate that formed was removed by suction filtration. The product was dried under vacuum and then recrystallized from chloroform to give compound 3 as white needles.

Yield (1.91 g, 78%), mp: 160 °C. ^1^H NMR (400 MHz, DMSO-d_6_): δ_H _9.63 (bs, 4H, ArOH), 6.85 (s, 8H, Ar-H), 4.25 (d, J = 12 Hz, 8H, ArCH_2_Ar, H_ax_), 3.27 (s, 8H, ArCH_2_N), 3.16 (d, J = 12 Hz, 8H, ArCH_2_Ar, H_eq_), 2.18 (s, 24H, NCH_3_); ^13^C NMR (100 MHz, DMSO-d_6_): δ_C_ 154.12 (ArC-O), 129.89 (C_(__o__)_ of Ar), 128.41 (C_(__m__)_ of Ar), 125.20 (ArC*-CH_2_), 62.49 (ArCH_2_N), 44.03 (NCH_3_), 32.51 (ArCH_2_Ar). Anal. Calcd for C_40_H_52_N_4_O_4_: C, 73.59; H, 8.03; N, 8.58. Found: C, 73.66; H, 7.96; N, 8.64. FAB ^+^ MS m/z = 652.37 (M ^+^).

*Amine quaternisation and eliminative nitrilation for the synthesis of compound 4*

To a solution containing compound 3 (1.63 g, 2.5 mmol) in DMSO (25 mL) was slowly added CH_3_I (1 mL, 15 mmol). After the reaction mixture was stirred for 30 min. at room temperature, NaCN (1.5 g, 30 mmol) was added, and the mixture was heated for 2 h at 80ºC in an atmosphere of N_2_. The solution was cooled, treated with ice water (100 mL), acidified with 2 N HCl, filtered, and air-dried. The crude product was recrystallized from CH_3_CN to yield compound 4 as a pale yellow solid.

Yield (1.28 g, 88%), mp > 414ºC. IR (KBr, ν, cm ^− 1^): 3140 (OH). 2245 (CN) ^1^H NMR (400 MHz, DMSO-d_6_): δ_H _10-9 (br s, 4H, OH), 7.04 (s, 8H, Ar-H), 3.89 (br s, 8H, ArCH_2_Ar), 3.74 (s, 8H, ArCH_2_CN); ^13^C NMR (100 MHz, DMSO-d_6_): δ_C_ 149.76 (ArC-O), 128.79 (C_(__o__)_ of Ar), 128.47 (C_(__m__)_ of Ar), 123.06 (ArC*–CH_2_), 119.33 (CN), 30.75 (ArCH_2_Ar), 21.62 (ArCH_2_CN). Anal. Calcd for C_36_H_28_N_4_O_4_: C, 74.48; H, 4.83; N, 9.66 Found: C, 74.41; H, 4.50; N, 9.58. FAB ^+^ MS m/z = 580.23 (M ^+^).

*Acidic hydrolysis for the synthesis of compound 5*

To compound 4 (1.15 g, 2 mmol), glacial acetic acid (20 mL), water (2 mL) conc. H_2_SO_4_ (3 mL) was added with stirring. The mixture refluxed for 6 h and then it was cooled and poured over ice water (100 mL) to precipitate after 18 h. The brownish precipitate was filtered, washed with cold water, dried in 80-90°C and triturated with MeOH (3 × 20 mL) to give compound 5 as white powder.

Yield (1.1 g, 84%), mp: 310-312 °C. IR (KBr, ν, cm ^− 1^): 3600-2500 (COO-H), 1750 (C = O). ^1^H NMR (400 MHz, DMSO-d_6_): δ_H _12.20 (b, 4H, CO_2_H), 9.60 (bs, 4H, ArOH), 6.95 (s, 8H, Ar-H), 3.82 (S, 8H, ArCH_2_Ar), 3.27(s, 8H, Ar-CH_2_CO_2_); ^13^C NMR (100 MHz, DMSO-d_6_): δ_C_ 172.8 (CO), 148.26 (ArC-O), 129.49 (C_(__o__)_ of Ar), 129.27 (C_(__m__)_ of Ar), 128.06 (ArC*-CH_2_), 38.52 (*CH_2_CO_2_H), 30.64 (ArCH_2_Ar). Anal. Calcd for C_36_H_32_O_12_: C, 65.85; H, 4.91. Found: C, 65.78; H, 4.50. FAB ^+^ MS m/z = 656.16 (M ^+^).

*Reduction of tetra acid for the synthesis of calixtyrosol 6*

LiAlH_4_ (3.4 g, 90 mmol) was added drop-wise to a solution of compound 5 (1 g, 1.5 mmol) in dry THF (100 mL) placed in an ice bath. The mixture was then heated under reflux for 24 h. The reaction mixture was cooled, then methanol (30 mL) was added and the solvents were evaporated under reduced pressure and finally the residue was washed twice by ice-water (50 mL). Recrystallization from MeCN gave compound 6 as colorless crystals.

Yield (0.6 g, 68%), mp: 358-360 °C. IR (KBr, ν, cm ^− 1^): 3237, 3182, 2930, 1452. ^1^H NMR (400 MHz, DMSO-d_6_, 50 °C): δ_H _11.08 (bs, 4H, OH), 9.61 (s, 4H, ArOH), 8.27 (s, 8H, ArH), 5.14 (s, 8H, ArCH_2_Ar), 4.83 (t, 8H, J = 7.2 Hz, ArCH_2_CH_2_*OH), 3.85 (t, 8H, J = 7.2 Hz, ArCH_2_*CH_2_OH); ^13^C NMR (100 MHz, DMSO-d_6_, 50 °C): δ_C_ 137.76 (ArC-O), 122.49 (C_(__o__)_ of Ar), 118.97 (C_(__m__)_ of Ar), 118.66 (ArC*–CH_2_), 52.42 (ArCH_2_Ar), 28.49 (ArCH_2_C*H_2_OH), 21.34 (ArC*H_2_CH_2_OH). Anal. Calcd for C_36_H_40_O_8_: C, 71.98; H, 6.71. Found: C, 72.06; H, 6.64. FAB ^+^ MS m/z = 600.19 (M ^+^).

*Antioxidant tests*

To a freshly prepared solution (2 mL) of DPPH (0.2 mM) in MeOH placed in the spectrometer cell was added a freshly prepared solution (20 μL) of antioxidant (2.5 mM) in MeOH. The reaction was monitored at 25 ºC over 10 minutes. Each experiment was repeated six times. Standard deviations were lower than 5%.

## Results and Discussion

*Chemistry*

**Scheme 1:**
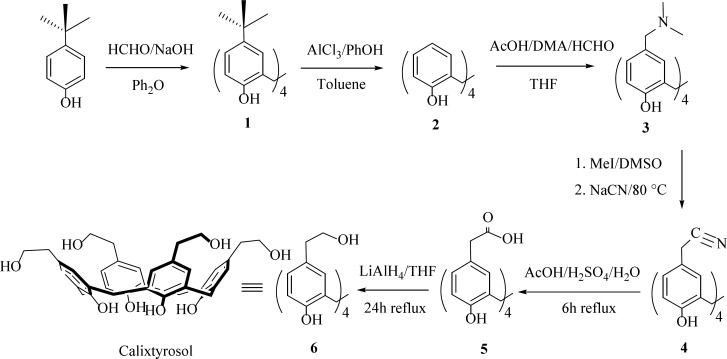
Synthetic pathway to calixtyrosol

The synthesis of the cluster 6 is depicted in Scheme 1. The synthetic strategy involves the grafting of hydroxyethyl moieties at the upper rim of the calix[4]arene platform via reduction of the corresponding tetraacid-calix[4]arene **5 **by using LiAlH_4_ in dry THF as solvent. 

The compound 5 was synthetized by a Mannich dimethylaminomethylation of calix[4]arene, quaternisation of amines followed by eliminative nitrilation and acidic hydrolysis of nitrile groups to the corresponding tetraacid-calix[4]arene. Their structures were confirmed by NMR spectra and elemental analysis.

*Quantitative radical scavenging test*

Free radical scavenging activity was determined by the 2, 2-diphenyl-1-picrylhydrazyl radical (DPPH^•^). DPPH is a stable nitrogen-centered free radical. A quantitative analysis of the Hydrogen atom transfer reaction from a certain antioxidant to DPPH reposes a very preferable way to delineate the antioxidant by the stoichiometric factor *n* tightly related to its intrinsic antioxidant activity (37). The H-transfer reactions are monitored by UV/VIS spectroscopy by recording the decline of the DPPH visible absorption band (λ_max _= 515 nm in MeOH) that is due to the conversion of the DPPH radical into the corresponding colorless hydrazine (DPPH–H) by the antioxidant. The experiments are run at a DPPH–antioxidant molar ratio of four in order to overhaul the H-donating power of the antioxidant. 

The solutions of antioxidants were prepared in MeOH. The stoichiometric factors *n* were calculated (with DPPH–Antioxidant molar ratio = 4) from the following equation:

*n* = (∆A_515_/ε_515_) /C where ∆A_515 _= A_0_ – A_f_ is the absorbance difference between the initial and stationary state of DPPH^• ^solution, ε_515 _= 11240 M ^– 1 ^cm ^– 1^, and C is the concentration of antioxidant in cuvette at time zero.

In order to evaluate the potentially enhanced radical scavenging of the multivalent compound 6, we compared it with tyrosol 7 as reference compound. In fact, tyrosol can be considered as 1/4 of the corresponding hybrid structure (Figure 1). For further comparison and appropriate verdict, we compared it with hydroxytyrosol 8 as an effective natural antioxidant and 3, 5-di-*tert*-butyl-4-hydroxytoluene (BHT) 9 as a synthetic antioxidant (Figure 1).

The stoichiometric factor *n* is the number of DPPH^•^ radicals quenched per antioxidant molecule and is demonstrator of radical inhibiting capability of antioxidant (Table 1).

**Table 1:** The stoichiometric factor *n *for reactions of Hydrogen atom transfer from certain antioxidant to DPPH (DPPH–Antioxidant molar ratio = 4, MeOH, 25°C).

**Antioxidant**	***n*** ** at 600 s**
Calixtyrosol	4.83
Tyrosol	---a
Hydroxytyrosol	2.45
BHT	2.63

a (–) Tyrosol reaction with DPPH• is very slow.

Antioxidant activity is a multifactorial event. This property in the phenolic compounds, depends on interest to radical formation, electron-donating substituents chemical stability, and etc.

Many synthetic antioxidants, which are characterized by a better antioxidant activity than natural antioxidants and contain mainly phenolic compounds whose structure allows them to form low-energy radicals through stable resonance hybrids and will not further propagate the oxidation reaction such as BHT (Table 1) (38).

Phenol itself does not act as an antioxidant, but substitution of bulky alkyl groups into *ortho*- and *para*-positions increase the electron density on the hydroxyl group by an inductive effect and thus enhances its reactivity toward lipid radicals (39).

*ortho*-Diphenols such as hydroxytyrosol (Figure 1) are more effective antioxidants than simple phenols (40), due to stabilization of the phenoxy-radical through hydrogen bonding (41).

The stability of the phenoxy radical is increased by bulky groups at the *ortho*-position, such as BHT (42). Since these substituents increase the steric hindrance in the region of the radicals, they further reduce the rate of possible propagation reactions that may occur involving antioxidant free radicals.

Overall, the presence of bulky branched groups in certain positions of antioxidant structure, hydrogen bonding and delocalization of the unpaired electron around the aromatic ring, increase the stability of phenoxy radicals. The stability of the phenoxy radicals reduces the rate of propagation and further reaction and thus increases the oxidative stability of lipids.

## Conclusion

In conclusion, the present work describes the first example of radical scavenging test of tyrosol–calixarene. This derivative could be considered as a high density antioxidant surface.

The results of the present study demonstrate a noteworthy increase in antioxidative property from the monomeric tyrosol to the corresponding cyclic tetramer. Presumably, it is attributed to tethering and arraying of the four impacted tyrosol, which make a synergistic effect in interactions with radicals for creating effective radical scavenging activity. In addition, probably, the presence of methylene bridges (*ortho*-position) as bulky groups, increasing the electron density on the hydroxyl group and hydrogen bonding at the lower rim of calixtyrosol are increased stabilization of the phenoxy-radical in the structure of antioxidant, which has a positive effect on reduction of the rate of possible propagation reactions.
